# Absorption Distribution Metabolism Excretion and Toxicity Property Prediction Utilizing a Pre-Trained Natural Language Processing Model and Its Applications in Early-Stage Drug Development

**DOI:** 10.3390/ph17030382

**Published:** 2024-03-17

**Authors:** Woojin Jung, Sungwoo Goo, Taewook Hwang, Hyunjung Lee, Young-Kuk Kim, Jung-woo Chae, Hwi-yeol Yun, Sangkeun Jung

**Affiliations:** 1 College of Pharmacy, Chungnam National University, Daejeon 34134, Republic of Korea; tnzo12@hotmail.com; 2 Department of Bio-AI convergence, Chungnam National University, Daejeon 34134, Republic of Korea; swgoo@outlook.kr (S.G.); hwangtw@o.cnu.ac.kr (T.H.); hjung0222@gmail.com (H.L.); 3 Computer Science and Engineering, Chungnam National University, Daejeon 34134, Republic of Korea

**Keywords:** machine learning, ADMET, drug discovery, in silico screening

## Abstract

Machine learning techniques are extensively employed in drug discovery, with a significant focus on developing QSAR models that interpret the structural information of potential drugs. In this study, the pre-trained natural language processing (NLP) model, ChemBERTa, was utilized in the drug discovery process. We proposed and evaluated four core model architectures as follows: deep neural network (DNN), encoder, concatenation (concat), and pipe. The DNN model processes physicochemical properties as input, while the encoder model leverages the simplified molecular input line entry system (SMILES) along with NLP techniques. The latter two models, concat and pipe, incorporate both SMILES and physicochemical properties, operating in parallel and with sequential manners, respectively. We collected 5238 entries from DrugBank, including their physicochemical properties and absorption, distribution, metabolism, excretion, and toxicity (ADMET) features. The models’ performance was assessed by the area under the receiver operating characteristic curve (AUROC), with the DNN, encoder, concat, and pipe models achieved 62.4%, 76.0%, 74.9%, and 68.2%, respectively. In a separate test with 84 experimental microsomal stability datasets, the AUROC scores for external data were 78% for DNN, 44% for the encoder, and 50% for concat, indicating that the DNN model had superior predictive capabilities for new data. This suggests that models based on structural information may require further optimization or alternative tokenization strategies. The application of natural language processing techniques to pharmaceutical challenges has demonstrated promising results, highlighting the need for more extensive data to enhance model generalization.

## 1. Introduction

Over the past few decades, the landscape of drug discovery has been significantly transformed by the integration of in silico methodologies, witnessing a substantial surge in efficiency and effectiveness. This revolution in computational approaches has been instrumental in streamlining the drug screening process, thereby offering the pharmaceutical industry considerable savings in terms of both costs and time. Among the various strategies employed, Quantitative Structure–Activity Relationship (QSAR) models have emerged as a cornerstone for predicting the chemical properties of compounds. The foundational premise of QSAR models is the assumption that compounds with analogous structures are likely to exhibit similar activities, thereby enabling the prediction of chemical activity through structural analysis.

Traditionally, QSAR models have relied on machine learning (ML) techniques, including but not limited to support vector machines, decision trees, naive Bayes, and k-nearest neighbors [1,2]. These methods typically dissect the structure of molecules into predefined molecular fragments or employ theoretical molecular descriptors, often determined through human judgment on a training dataset. Such an approach, while functional, has its limitations, particularly in terms of predictability on novel datasets.

However, the advent of deep learning is reshaping this landscape by addressing the shortcomings of conventional QSAR methodologies. Deep learning algorithms have the capacity to algorithmically define the criteria for analysis, thus bypassing the constraints imposed by human-set parameters. This advancement not only enhances the predictive accuracy of these models but also broadens their application. Furthermore, a significant limitation of traditional QSAR models has been their reliance solely on compounds with available ADMET experimental results for model construction. Considering the vast number of synthesized compounds, the subset with ADMET data is relatively small, posing a considerable challenge to the generalization of ADMET prediction models.

An online competition held in 2012 revealed the potential of deep learning algorithms to address problems with pharmaceuticals, such that there has been a shift toward the deep-learning techniques. Although deep learning has shown promising results that can replace traditional methods, some problems in deep learning remain [3]. Deep learning models tend to improve their performance by memorizing the inputs, which can increase their dependency on the tested data [4,5,6]. This tendency is even more pronounced in pharmaceutical fields, for example, the relationship between a molecular structure and its properties. Because molecular data come in various forms depending on their specific domain, many efforts to generate compatible data and to make a link between various domains are under process. Due to the mutual understanding of computer-aided drug design (CADD) in pharmaceutical fields, various trials to predict pharmacologic features and endpoints in drug development are being made with machine learning [7,8,9]. In terms of pharmacology, the features concerning absorption, distribution, metabolism, excretion, and toxicity (ADMET) are of significant interest in typical drug development and can be used for weighing the systemic exposure and potential side effects of a candidate drug. Since this systemic exposure is affected by numerous factors and features, reliable ADMET prediction has the utmost priority before candidate drugs are further evaluated in real clinical situations [10].

In techniques for deep learning, graph convolutional neural networks (GCNNs) enable the dynamic learning of chemical structures by considering a space for atoms and adjacent bonds [11]. After the advantages of GCNNs were demonstrated, new featurization approaches based on multitasking or sequential learning were implemented using GCNNs, leading to further performance improvements [12]. However, despite these improvements, GCNNs have difficulties with unlabeled structures because they require many feature parameters. In a recent study, however, contrastive learning in GCNN was introduced to resolve this problem [13], showing remarkable performance improvements along the tasks. Just as there was a certain level of progression in the performance utilizing graph neural networks, this was also demonstrated in natural language processing. Transformer-based learning is vigorously performed in this field; recent natural language technique applications during drug development tasks were successful in improving benchmark results. Within natural language processing (NLP), bidirectional encoder representations from transformers (BERT) have significantly improved NLP over the past 4 years via transformer pre-training and task-specific model fine-tuning [14]. Because BERT is generally used in conjunction with masked language modeling (MLM), it is also expected to be able to deal with the atom, masking the problems seen in GCNNs.

In addition, BERT is capable of handling large amounts of data because it was originally designed to deal with large volumes of text. In 2020, Chithrananda et al. introduced ChemBERTa, which contains 77 million simplified molecular input-line entry systems (SMILES) from PubChem and was designed to perform large-scale self-supervised pre-training for molecular property predictions. ChemBERTa is expected to provide promising performance for representation learning and molecular property prediction as a pre-trained model [15].

In addition to BERT, models that employ transformers and that have shown effectiveness in masked modeling, such as BART (Bidirectional Auto-Regressive Transformers) [16] and ELECTRA (Efficiently Learning an Encoder that Classifies Token Replacements Accurately) [17], could serve as promising pre-trained models in drug discovery. Performance metrics in these studies have exceeded those of traditional approaches in many tasks, as has been previously demonstrated in language tasks [18].

Both GCNN and NLP models are continuously evolving, complementing each other’s weaknesses, yet an examination in terms of the weaknesses and strengths of the NLP technique is not sufficiently considered across the aspects of pharmacy. In this study, (1) the performance of natural language models of ChemBERTa and ELECTRA were assessed on benchmark datasets with other prediction models, and (2) large-scale transfer learning with fine tunings to natural language models in ADMET problems was carried out to test its ability to perform multi-task prediction. (3) The models were then assessed on the external dataset to investigate the NLP model’s generalization towards ADMET problems.

## 2. Results

### 2.1. MoleculeNet Dataset

In Table 1 and Table 2, the results from the MoleculeNet dataset are shown. The mean and standard deviation of AUROC or RMSE and MAP on each dataset are reported. In reference to the benchmark result from Wang et al., the performance metrics of supervised learning models or graph models were compared with those of ChemBERTa and ELECTRA. In classification tasks, ChemBERTa recorded around mid-ranks on average and showed superior performance on toxicity problems like Tox21 and ClinTox (ranked 1st and 3rd, respectively). ELECTRA ranked slightly below ChemBERTa scores in general. Both ChemBERTa and ELECTRA scored almost the lowest in regression tasks. The performance of ELECTRA was lower than that of ChemBERTa in most tasks except ESOL and QM7.

### 2.2. DrugBank Dataset

Based on the AUROC values, the encoder model had the best performance (76.0%), followed by the concat model (74.9%), the pipe model (68.2%), the DNN_A model (63.6%), the DNN model (62.4%), and the pipe_A model (61.2%). The encoder and concat models, which included pre-trained models, generally showed higher predictive power than the others. The pipe model showed comparatively low performance, even though it utilized a pre-trained model. The incorporation of attention slightly increased the performance of the DNN model but decreased the pipe model’s performance.

Although DNN is the simplest model, it uses parameters that are considered important in drug development, and it can be identified that the performance is not significantly inferior compared to other models. It was shown that the performance of the DNN’s simple model slightly improved due to the addition of attention. Considering that encoder and concat are similar in structure and differ only in the input information, it has been shown that important structural information can be sufficiently reflected by SMILES in the QSAR work process.

**Figure 1:**
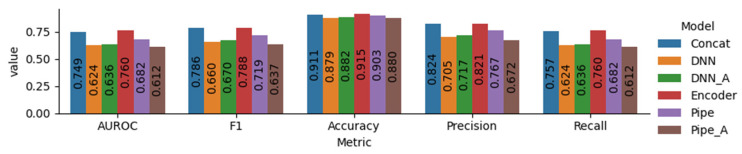
Performance metrics of total features (Table A1) for suggested models.

**Figure 2:**
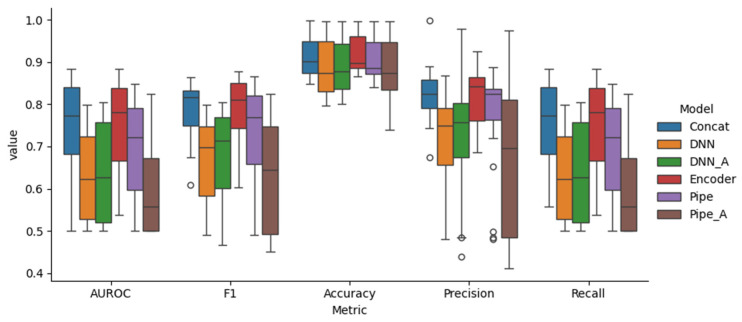
Box plots of the distribution of performance metrics for each feature (Table A3) of suggested models.

The pipe model, comprising two steps that predict physicochemical information and ADMET properties, may have exhibited decreased performance due to uncertainties introduced during the learning processes. This issue was likely more pronounced in the pipe model, which utilized the attention algorithm, adding complexity. A summary of the performance of the output label is described in Figure 1 and Figure 2. 

### 2.3. External Dataset

The DNN, encoder, and concat models had AUROC values of 0.78, 0.44, and 0.50, respectively. When tested only with CYP 3A4 substrate prediction, the matched label proportions for the test data were 0.631, 0.583, and 0.571. For weighted soft voting, which analyzes the abundance of CYP450 subtype enzymes, the matched label proportions for the test data were 0.619, 0.571, and 0.583, respectively. In all three assessment methods, DNN scored the best.

### 2.4. Applicability Domain

The models were developed using datasets from PubChem and DrugBank. Initially, the PubChem dataset was employed for the pre-training of the language model through MLM techniques. This step enabled the model to understand the structures of a wide variety of substances. The models were then fine-tuned with the DrugBank dataset, with a focus on the ADMET features of substances classified as drugs. The scope of chemical structures targeted by these models was those cataloged in PubChem. To assess the model’s applicability and its limitations within this domain, the external dataset—comprising rates of CYP450 enzyme reactions for toxic substances not listed in DrugBank—was employed for validation purposes. The validation showed that the DNN model, which is close to traditional QSAR models, had superior performance, but within the DrugBank dataset, other models performed better. This suggests that these models are more adept at predicting the ADMET features of therapeutic drugs rather than toxic substances.

## 3. Discussion

In the MoleculeNet benchmark dataset, the pre-trained NLP models generally exhibited good performance in classification tasks. However, in most regression tasks, the NLP models demonstrated poor performance, with other models surpassing the NLP model metrics, especially in tasks predicting physicochemical properties. It is believed that the regression tasks require more detailed information on atomic spacing, which the NLP models used in this study cannot fully consider. On the other hand, the classification tasks resulted in better outcomes with simpler model implementations. In the Tox21 dataset, the NLP models achieved a better AUROC (82.3% and 80%, respectively) compared to the latest GNN techniques. The lower performance of ELECTRA in this study, compared to BERT in previous studies, could be attributed to its pre-training on a smaller set of molecules. It is anticipated that ELECTRA’s performance will improve with further pre-training using SMILES information. Moreover, as GNN techniques have enhanced their performance by addressing atom-masking tasks, these NLP models could also see improved performance by developing an approach that considers the precise functional space or by introducing another tokenization method to generate the minimal unit of functional atom groups.

The DNN model resembles the traditional QSAR model. Similar to its predecessor, it trains exclusively on datasets containing results from ADMET experiments without employing a pre-training approach. In contrast, other models, such as encoder, concat, and pipe, utilize a fine-tuning strategy with pre-trained NLP models. Except for pipe_A, these models demonstrated superior performance compared to the DNN. This outcome validates the efficacy of the NLP’s MLM training technique in capturing the structural nuances of chemical compounds.

In an effort to enhance model accuracy, we explored the concat model and pipe model, which integrate SMILES notation alongside the physicochemical properties of compounds. However, the encoder model, relying solely on SMILES notation, emerged as the most effective. This can be attributed to the fact that pharmaceutical development typically focuses on compounds adhering to specific physicochemical criteria, such as Lipinski’s rule of five and the Ghose filter. The limited variance in physicochemical properties within the training dataset presumably had minimal impact on the model performance.

When assessed using the external dataset, the DNN model’s performance surpassed its counterparts (the concat and encoder models). The external dataset comprised toxic compounds, which often deviate from conventional guidelines like Lipinski’s rule of five. This deviation suggests that the range of physicochemical properties in the training dataset is considerably narrower than that in the external dataset. This finding underscores the significance of employing diverse and unbiased datasets to bolster the model’s generalization capability.

## 4. Materials and Methods

### 4.1. Data Collection and Preprocessing

Three datasets were collected to evaluate the learning under different conditions. To derive quantitative benchmark results in comparison to other machine learning techniques, the dataset from MoleculeNet was used [18]. The data from DrugBank [38] was used to assess the model’s schematic position during drug development steps. Among the labels used in learning in the DrugBank dataset, we created an external dataset (unseen in the learning process) to evaluate the model trained on the DrugBank data.

#### 4.1.1. MoleculeNet Dataset

To assess the performance of the model on classification and regression problems, datasets from MoleculeNet were used [39]. A total of 13 datasets were selected for benchmarking, consisting of 44 binary classification tasks and 24 regression tasks. The datasets of BBBP (Blood–Brain Barrier Penetration) [19], Tox21 (Toxicology in the 21st Century) [20], ClinTox (clinical trial toxicity) [21], HIV (AIDS Antiviral Screen Data) [22], BACE (beta-site APP cleaving enzyme 1) [23], SIDER (Side Effect Resource) [24], and MUV (Maximum Unbiased Validation) [25] were chosen for classification tests. For regression tests, the datasets of FreeSolv (Database of Experimental and Calculated Hydration Free Energies) [33], ESOL (Estimating Aqueous Solubility) [34], Lipo (Experimental in vitro DMPK and physicochemical data on a set of publicly disclosed compounds) [35], QM7 (quantum-machine 7) [36], QM8 [37], and QM9 [37] were chosen. The chosen datasets cover various domains, including physiology, biophysics, physical chemistry, and quantum mechanics, coupled with molecular SMILES information. The benchmarks were compared with known prediction models and GNN-based techniques. As a reference for GNN models, the results of Wang et al. were used [13].

#### 4.1.2. DrugBank Dataset

Datasets for training, testing, and validation were collected from DrugBank. We obtained 13,856 raw JSON files. Each file contained drug information such as the name, description, attribute values, related molecules, and applications. In total, 5238 raw files contained SMILES and ADMET data, and 18 features extracted from the ”Experimental Properties” and ”Predicted Properties” tabs were used for model training as follows: SMILES, LogP, LogS, pKa, water solubility, physiological charge, hydrogen acceptor count, hydrogen donor count, polar surface area, rotatable bond count, molar refractivity, polarizability, the number of rings, bioavailability, and drug-likeness filters including Lipinski’s rule of five [40], the Ghose filter [41], Veber’s rule [42], and the MDDR-like rule [43] (Table A3). The filter properties of bioavailability, Lipinski’s rule of five, the Ghose filter, Vebers’ rule, and MDDR-like rule are Boolean-type data that determine whether the information is ‘true or false’, and, for all other properties except the SMILES, it meant that molecular formulas were numeric data types. Among the extracted data, the four filter values of Veber’s rule, the MDDR-like rule, Lipinski’s rule of five, and the Ghose filter were excluded since those values could not be determined from the experiment, and possible overfitting was observed in the pre-test. The models were used to predict 21 ADMET features extracted from the “Predicted ADMET Features” Table, and these features are described in Table A3. To avoid semantic redundancy in the outputs, the labels of human intestinal absorption and Caco-2 permeability were combined into human intestinal absorption. Two p-glycoprotein inhibitor (I and II) descriptors were combined into one p-glycoprotein inhibitor, and the same was performed for the two hERG inhibition descriptors. If one of the 18 features was missing from a raw file, the corresponding chemical was excluded from the analysis.

#### 4.1.3. External Dataset

Additional model testing was performed using 84 compounds externally collected by the CYP assay to evaluate the ability to perform CYP substrate prediction. The external dataset included the chemical structure, formula, and metabolism of CYP in human liver microsomes. These chemical structures were encoded in the SMILES format, and the classification of a compound as a CYP substrate was determined by the percentage of the chemical that remained following a specified reaction duration.

### 4.2. Deep Learning Models

**Figure 3:**
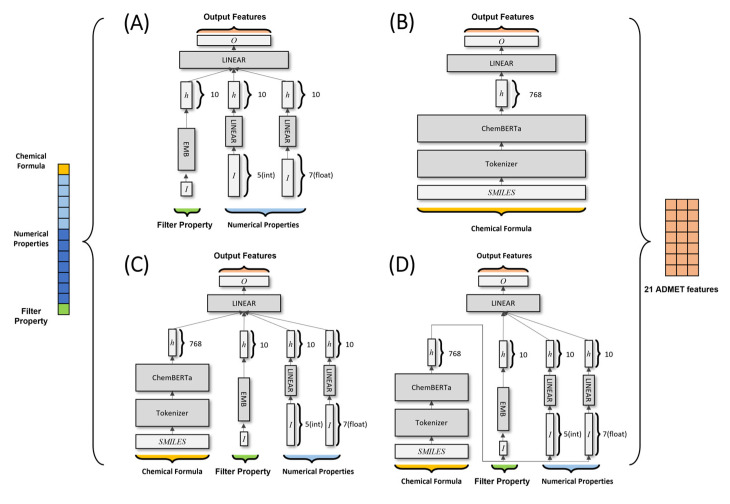
Schematic flow of prepared core models, (**A**): DNN, (**B**): encoder, (**C**): concat, and (**D**): pipe. Single SMILES and Boolean features, five integers, and seven float values are concatenated and processed in the model before the values are transformed into 21 output values. I: input, h: hidden layer, EMB: embedding layer, LINEAR: full-connected layer.

For the MoleculeNet dataset, both ChemBERTa and ELECTRA were utilized in the benchmarks. For tokenization, a byte pair encoder (BPE) [44]-based SMILES tokenizer and WordPiece were used, respectively [15,45]. BPE is a sub-word level tokenization technique that processes the maximum number of words in a text corpus. Given the unlimited number of letter combinations, even unknown words can be processed by decomposing them into multiple-letter combinations. Thus, even SMILES can be expressed as a set of sub-SMILES. WordPiece Tokenizer is a variant algorithm of BPE. The algorithm merges the pairs with the highest ’likelihood’ of the corpus when merged, as opposed to merging the pairs in which the BPE appears most frequently based on their ’frequency’. The ELECTRA model pays more attention to the efficiency of learning as well as the accuracy of the model. ELECTRA includes new pre-training tasks called Replaced Token Detection (RTD) to improve learning efficiency, through which ELECTRA learns faster and more effectively. For this study, we used ELECTRA-small and randomly extracted 10 M molecules that were pre-trained from the PubChem 109 M dataset with 10 epochs. For the DrugBank dataset, an NLP model with better performance in the benchmark was selected and used in learning. Six models with different structures were tested in this study (Figure 3); they were all trained using a cross-entropy loss function.

1. A deep neural network (DNN) model consists of fully connected and embedded layers. The model uses 18 input physicochemical values as input properties. One Boolean feature was transformed into a 10-dimensional vector via embedding layers. Five integer-based and seven float features were transformed into a 10-dimensional vector via fully connected layers. Vectors were concatenated into a 30-dimensional vector, which passed through fully connected layers to return a vector with 21 dimensions (the predicted ADMET features).
2. An encoder model includes a pre-trained ChemBERTa model. This model treats SMILES data as “natural sentences” and learns via MLM, which is RoBERTa. The SMILES data used for the pre-trained model are in the form of a 768-dimensional hidden vector, which is transformed into an 18-dimensional vector via fully connected layers.
3. A concat model combines the DNN and encoder models described above. The 30-dimensional vector from the DNN model and the 768-dimensional hidden vector from the encoder model are concatenated and passed to the hidden layer of the concat model. This 798-dimensional hidden vector is then transformed into 21 dimensions.
4. A pipe model, which subsumed a pre-trained ChemBERTa model, used a 768-dimensional hidden vector based on SMILES data to predict 21 physicochemical properties. Those physicochemical properties were then used as input for a DNN model to predict ADMET features.
5. A modified version of the DNN model is DNN A (where A stands for attention). We incorporated dot-product self-attention into the model, which uses hidden vectors from the DNN as the query, key, and value. By implementing dot-product self-attention, it was possible to identify which input most affected ADMET feature predictions.
6. A modified version of the pipe model is pipe A, into which dot-product self-attention can be incorporated.

### 4.3. Settings

The data were divided into training, validation, and test sets (ratio of 7:1.5:1.5). All numeric data were normalized. The batch size was fixed at 32, with the learning rate at 5 × 10^−5^. Early stopping was used during training; this automatically terminated training if the validation loss value did not drop for five epochs. A maximum of 30 epochs were allowed before early stopping. For more accurate performance measurements, each model was constructed five times, with identical parameters using different random seeds for generating random numbers during model initialization. As an optimizer, AdamW was used. For each classification and regression problem, binary cross entropy and mean squared errors (MSEs) were used for the loss function. No activation function was used except in the case of attention models, which used the tanh activation model. Model training was performed using a computer with an Nvidia A100 GPU, AMD EPYC ROME 7742 CPU, and 1 TB of RAM.

### 4.4. Evaluation

In benchmark datasets, model evaluation was performed in the area under the receiver operating characteristic curve (AUROC) for classification tasks. For regression tasks, FreeSolv, ESOL, and Lipo used the root mean square error (RMSE), while QM7, QM8, and QM9 were measured with the mean absolute error (MAE) in accordance with MoleculeNet’s recommendation. Performance evaluation in the DrugBank dataset was based on accuracy, the area under the receiver operating characteristic curve (AUROC), F1, precision, and recall. Accuracy refers to the proportion of data correctly predicted by the model. Accuracy is an intuitive metric, but these data should be balanced to evaluate accuracy appropriately. For example, if a test dataset consists of 100 values, of which 99 are true values and 1 is a false value, the accuracy would be 99% if the model predicted 100 true values without any conditions, which implies that the metric is biased. When these data are unbalanced, it is difficult to obtain reliable results. Precision, recall, and F1 should be included as performance metrics to overcome potential bias in the accuracy evaluation. Precision refers to the proportion of actual true values relative to all predicted true values. Precision evaluation does not take predicted false values into account; only true values are considered, which gives rise to bias. Therefore, precision alone is not a reliable metric. Recall refers to the proportion of true values correctly predicted by the model. This metric does not take predicted false values into account. The problem with recall is that if all values are predicted to be true, performance is considered perfect. Recall and precision are related; if precision increases, recall tends to decrease.

Precision and recall are good metrics when several conditions are satisfied, but due to their bias, F1 (the harmonic average of precision and recall) is the most used metric. Precision and recall are complementary, but both values must be high for F1 to be high; thus, F1 represents a compromise that solves the problems of precision and recall. The receiver operating characteristic (ROC) curve can show the predictive performance of a model at different thresholds. ROC curves plot recall against specificity, which is also complimentary. Specificity is defined as the proportion of false values predicted correctly by the model. The AUROC is a commonly used metric that increases when a model predicts both true and false values accurately; therefore, it has good evaluation performance. The prediction results of the six models used in this experiment were voted on: when the result was a tie (3:3), the highest average prediction probability of each model was considered the final value.

When evaluating the external data, the CYP450 substrate prediction performance was assessed with the DNN, encoder, and concat models. Since the label did not match with what was predicted from the models, performance evaluation was conducted on the following three methods of transformation. (1) The CYP450 subtypes’ substrate-predicted value for each model was concatenated into one vector and then synthesized into one feature of the CYP substrate (logical, true, or false) with deep neural networks. (2) The CYP 3A4 (the major enzyme in CYP metabolism) substrate value was taken directly for the CYP substrate. (3) The prediction was compared to the results of weighted soft voting regarding CYP450 abundance. Abundance was set to 12% for the CYP450 subtype 2C9, 4% for subtype 2D6, and 30% for subtype 3A4. In the case of (1), the performance was measured by the AUROC, and for the rest of the method, performance was measured in matched proportion with the test data.

## 5. Conclusions

In traditional ADMET prediction models, the scope was narrowly confined to compounds with pre-existing experimental ADMET data. This limitation significantly curtailed the models’ generalizability, as the dataset of compounds with known ADMET outcomes was substantially smaller than the entire pool of synthesized compounds. Additionally, these conventional models often introduced bias by incorporating human-defined molecular fragments or theoretical molecular descriptors. In contrast, NLP (Natural Language Processing) models have adopted the strategy of unsupervised pre-training on extensive datasets, which is a technique proven to bolster model performance while also reducing the potential for human-induced biases. However, when evaluated against the external dataset, simpler models, such as the DNN model, outperformed more complex ones. This discrepancy unveiled a decline in performance when dealing with heterogeneous datasets, suggesting that generalization capabilities might be compromised due to dataset bias. Enhancing our dataset with a more diverse array of data points could, therefore, further refine the accuracy of deep learning models. To improve model robustness and lessen reliance on large datasets, we advocate for methodological advancements, including data augmentation, few-shot learning, and the adoption of sophisticated pre-trained models proficient in interpreting the SMILES notation.

## Figures and Tables

**Table 1:** Mean and standard deviation (in parenthesis) of AUROC measures on 7 classification benchmarks. Supervised learning models: first seven rows. Self-supervised/pre-training methods: rows eight to thirteen. Tested models (ChemBERTa and ELECTRA): rows twelve and thirteenth. (RF: random forest. SVM: support vector machine. #: Number of).

Dataset	BBBP [19]	Tox21 [20]	ClinTox [21]	HIV [22]	BACE [23]	SIDER [24]	MUV [25]
# Molecules	2039	7831	1478	41127	1513	1427	93,087
# Tasks	1	12	2	1	1	27	17
RF	71.4 (0.0)	76.9 (1.5)	71.3 (5.6)	78.1 (0.6)	86.7 (0.8)	68.4 (0.9)	63.2 (2.3)
SVM	72.9 (0.0)	81.8 (1.0)	66.9 (9.2)	79.2 (0.0)	86.2 (0.0)	68.2 (1.3)	67.3 (1.3)
GCN [26]	71.8 (0.0)	70.9 (2.6)	62.5 (2.8)	74 (3.0)	71.6 (2.0)	53.6 (3.2)	71.6 (4.0)
GIN [27]	65.8 (4.5)	74 (0.8)	58 (4.4)	75.3 (1.9)	70.1 (5.4)	57.3 (1.6)	71.8 (2.5)
SchNet [28]	84.8 (2.2)	77.2 (2.3)	71.5 (3.7)	70.2 (3.4)	76.6 (1.1)	53.9 (3.7)	71.3 (3.0)
MGCN [29]	85 (6.4)	70.7 (1.6)	63.4 (4.2)	73.8 (1.6)	73.4 (3.0)	55.2 (1.8)	70.2 (3.4)
D-MPNN [30]	71.2 (3.8)	68.9 (1.3)	90.5 (5.3)	75 (2.1)	85.3 (5.3)	63.2 (2.3)	76.2 (2.8)
Hu et al. [31]	70.8 (1.5)	78.7 (0.4)	78.9 (2.4)	80.2 (0.9)	85.9 (0.8)	65.2 (0.9)	81.4 (2.0)
N-Gram [32]	91.2 (3.0)	76.9 (2.7)	85.5 (3.7)	83 (1.3)	87.6 (3.5)	63.2 (0.5)	81.6 (1.9)
MolCLR-GCN [13]	73.8 (0.2)	74.7 (0.8)	86.7 (1.0)	77.8 (0.5)	78.8 (0.5)	66.9 (1.2)	84 (1.8)
MolCLR-GIN [13]	73.6 (0.5)	79.8 (0.7)	93.2 (1.7)	80.6 (1.1)	89 (0.3)	68 (1.1)	88.6 (2.2)
ChemBERTa	73.4 (1.4)	82.3 (0.9)	88.9 (3.6)	74.5 (3.1)	79.2 (2.0)	60.4 (2.0)	73.9 (3.4)
ChemELECTRA	72.5 (2.0)	80 (1.0)	84.6 (3.3)	73.7 (2.9)	76.9 (2.5)	56.9 (1.8)	73.7 (2.8)

**Table 2:** Mean and standard deviation (in parenthesis) of RMSE and MAE measures. RMSE for FreeSolv, ESOL, and Lipo dataset; MAE for QM7, QM8, and QM9. Supervised learning models: first seven rows. Self-supervised/pre-training methods: rows eight to thirteen. Tested models (ChemBERTa and ELECTRA): rows twelve and thirteen (RF: random forest. SVM: support vector machine. #: Number of).

Dataset	FreeSolv [33]	ESOL [34]	Lipo [35]	QM7 [36]	QM8 [37]	QM9 [37]
# Molecules	642	1128	4200	6830	21,786	130,829
# Tasks	1	1	1	1	12	8
RF	2.03 (0.22)	1.07 (0.19)	0.88 (0.04)	122.7 (4.2)	0.0423 (0.0021)	16.061 (0.019)
SVM	3.14 (0.0)	1.5 (0.0)	0.82 (0.0)	156.9 (0.0)	0.0543 (0.001)	24.613 (0.144)
GCN	2.87 (0.14)	1.43 (0.05)	0.85 (0.08)	122.9 (2.2)	0.0366 (0.0011)	5.796 (1.969)
GIN	2.76 (0.18)	1.45 (0.02)	0.85 (0.07)	124.8 (0.7)	0.0371 (0.0009)	4.741 (0.912)
SchNet	3.22 (0.76)	1.05 (0.06)	0.91 (0.1)	74.2 (6)	0.0204 (0.0021)	0.081 (0.001)
MGCN	3.35 (0.01)	1.27 (0.15)	1.11 (0.04)	77.6 (4.7)	0.0223 (0.0021)	0.05 (0.002)
D-MPNN	2.18 (0.91)	0.98 (0.26)	0.65 (0.05)	105.8 (13.2)	0.0143 (0.0022)	3.241 (0.119)
Hu et al. [31]	2.83 (0.12)	1.22 (0.02)	0.74 (0.0)	110.2 (6.4)	0.0191 (0.0003)	4.349 (0.061)
N-Gram	2.51 (0.19)	1.1 (0.03)	0.88 (0.12)	125.6 (1.5)	0.032 (0.0032)	7.636 (0.027)
MolCLR-GCN	2.39 (0.14)	1.16 (0.0)	0.78 (0.01)	83.1 (4.0)	0.0181 (0.0002)	3.552 (0.041)
MolCLR-GIN	2.2 (0.2)	1.11 (0.01)	0.65 (0.08)	87.2 (2.0)	0.0174 (0.0013)	2.357 (0.118)
ChemBERTa	5 (0.11)	2.06 (0.02)	1.2 (0.0)	187.7 (2.7)	0.0333 (0.0003)	20.941 (0.199)
ChemELECTRA	5.03 (0.13)	2.05 (0.0)	1.2 (0.0)	179.1 (0.7)	0.0359 (0.0002)	24.228 (0.314)

## Data Availability

Data is contained within the article.

## Appendix A. Supplementary Results

**Table A1:** Total performance metrics.

Feature	Model	AUROC	F1	Accuracy	Precision	Recall
Ames test	Concat	0.679	0.730	0.901	0.790	0.679
	DNN	0.504	0.583	0.878	0.689	0.504
	DNN_A	0.500	0.467	0.878	0.439	0.500
	Encoder	0.655	0.700	0.891	0.751	0.655
	Pipe	0.510	0.614	0.879	0.773	0.510
	Pipe_A	0.500	0.467	0.878	0.439	0.500
Biodegradation	Concat	0.840	0.853	0.917	0.867	0.840
	DNN	0.749	0.771	0.875	0.795	0.749
	DNN_A	0.757	0.779	0.879	0.802	0.757
	Encoder	0.854	0.857	0.917	0.860	0.854
	Pipe	0.806	0.821	0.899	0.835	0.806
	Pipe_A	0.500	0.452	0.823	0.412	0.500
Blood–Brain Barrier	Concat	0.845	0.832	0.868	0.820	0.845
	DNN	0.624	0.697	0.798	0.791	0.624
	DNN_A	0.677	0.715	0.805	0.757	0.677
	Encoder	0.835	0.844	0.885	0.854	0.835
	Pipe	0.757	0.804	0.861	0.857	0.757
	Pipe_A	0.669	0.733	0.818	0.811	0.669
Caco-2 permeable	Concat	0.792	0.828	0.868	0.867	0.792
	DNN	0.772	0.783	0.831	0.794	0.772
	DNN_A	0.776	0.790	0.837	0.804	0.776
	Encoder	0.859	0.865	0.893	0.872	0.859
	Pipe	0.768	0.796	0.845	0.827	0.768
	Pipe_A	0.624	0.644	0.739	0.665	0.624
Carcinogenicity	Concat	0.607	0.690	0.973	0.801	0.607
	DNN	0.500	0.493	0.971	0.485	0.500
	DNN_A	0.500	0.493	0.971	0.485	0.500
	Encoder	0.627	0.688	0.972	0.762	0.627
	Pipe	0.520	0.579	0.969	0.653	0.520
	Pipe_A	0.500	0.493	0.971	0.485	0.500
CYP450 1A2 substrate	Concat	0.810	0.823	0.872	0.836	0.810
	DNN	0.722	0.747	0.823	0.773	0.722
	DNN_A	0.758	0.777	0.841	0.796	0.758
	Encoder	0.782	0.811	0.866	0.843	0.782
	Pipe	0.791	0.821	0.873	0.853	0.791
	Pipe_A	0.782	0.804	0.860	0.826	0.782
CYP450 2C19 inhibitor	Concat	0.762	0.788	0.884	0.816	0.762
	DNN	0.575	0.637	0.831	0.713	0.575
	DNN_A	0.577	0.632	0.828	0.699	0.577
	Encoder	0.780	0.802	0.891	0.825	0.780
	Pipe	0.722	0.770	0.879	0.824	0.722
	Pipe_A	0.664	0.716	0.856	0.777	0.664
CYP450 2C9 inhibitor	Concat	0.759	0.764	0.903	0.769	0.759
	DNN	0.528	0.570	0.874	0.619	0.528
	DNN_A	0.515	0.608	0.883	0.742	0.515
	Encoder	0.751	0.754	0.898	0.756	0.751
	Pipe	0.673	0.737	0.907	0.814	0.673
	Pipe_A	0.514	0.547	0.874	0.585	0.514
CYP450 2C9 substrate	Concat	0.500	0.800	0.997	0.999	0.667
	DNN	0.500	0.499	0.996	0.498	0.500
	DNN_A	0.500	0.499	0.996	0.498	0.500
	Encoder	0.666	0.705	0.996	0.749	0.666
	Pipe	0.500	0.499	0.996	0.498	0.500
	Pipe_A	0.500	0.499	0.996	0.498	0.500
CYP450 2D6 inhibitor	Concat	0.616	0.673	0.949	0.743	0.616
	DNN	0.584	0.698	0.954	0.867	0.584
	DNN_A	0.610	0.752	0.959	0.979	0.610
	Encoder	0.669	0.765	0.962	0.894	0.669
	Pipe	0.608	0.722	0.957	0.888	0.608
	Pipe_A	0.500	0.487	0.948	0.474	0.500
CYP450 2D6 substrate	Concat	0.698	0.782	0.973	0.888	0.698
	DNN	0.500	0.490	0.962	0.481	0.500
	DNN_A	0.533	0.644	0.963	0.815	0.533
	Encoder	0.664	0.743	0.969	0.844	0.664
	Pipe	0.500	0.490	0.962	0.481	0.500
	Pipe_A	0.500	0.490	0.962	0.481	0.500
CYP450 3A4 inhibitor	Concat	0.738	0.750	0.874	0.761	0.738
	DNN	0.535	0.590	0.842	0.656	0.535
	DNN_A	0.520	0.588	0.845	0.675	0.520
	Encoder	0.758	0.764	0.879	0.771	0.758
	Pipe	0.654	0.704	0.868	0.763	0.654
	Pipe_A	0.558	0.620	0.847	0.696	0.558
CYP450 3A4 substrate	Concat	0.870	0.864	0.879	0.858	0.870
	DNN	0.755	0.766	0.803	0.778	0.755
	DNN_A	0.766	0.769	0.802	0.772	0.766
	Encoder	0.838	0.850	0.873	0.863	0.838
	Pipe	0.826	0.827	0.850	0.828	0.826
	Pipe_A	0.786	0.786	0.814	0.786	0.786
CYP450 inhibitory promiscuity	Concat	0.843	0.832	0.874	0.822	0.843
	DNN	0.723	0.736	0.818	0.750	0.723
	DNN_A	0.727	0.729	0.807	0.732	0.727
	Encoder	0.854	0.848	0.888	0.842	0.854
	Pipe	0.821	0.826	0.877	0.832	0.821
	Pipe_A	0.786	0.799	0.860	0.813	0.786
hERG inhibition (precisiondictor I)	Concat	0.557	0.610	0.966	0.673	0.557
	DNN	0.500	0.492	0.968	0.484	0.500
	DNN_A	0.500	0.492	0.968	0.484	0.500
	Encoder	0.538	0.603	0.967	0.685	0.538
	Pipe	0.500	0.492	0.968	0.484	0.500
	Pipe_A	0.500	0.492	0.968	0.484	0.500
hERG inhibition (precisiondictor II)	Concat	0.835	0.830	0.905	0.825	0.835
	DNN	0.641	0.688	0.854	0.743	0.641
	DNN_A	0.690	0.724	0.864	0.762	0.690
	Encoder	0.821	0.828	0.907	0.836	0.821
	Pipe	0.746	0.775	0.885	0.806	0.746
	Pipe_A	0.743	0.767	0.880	0.793	0.743
Human intestinal absorption	Concat	0.884	0.854	0.896	0.827	0.884
	DNN	0.727	0.771	0.868	0.820	0.727
	DNN_A	0.767	0.795	0.878	0.824	0.767
	Encoder	0.883	0.878	0.921	0.873	0.883
	Pipe	0.848	0.867	0.919	0.887	0.848
	Pipe_A	0.615	0.693	0.835	0.795	0.615
P-glycoprotein inhibitor I	Concat	0.780	0.816	0.916	0.856	0.780
	DNN	0.634	0.703	0.878	0.789	0.634
	DNN_A	0.711	0.744	0.885	0.780	0.711
	Encoder	0.800	0.828	0.920	0.857	0.800
	Pipe	0.736	0.782	0.903	0.835	0.736
	Pipe_A	0.672	0.748	0.893	0.845	0.672
P-glycoprotein inhibitor II	Concat	0.683	0.716	0.894	0.753	0.683
	DNN	0.577	0.629	0.879	0.692	0.577
	DNN_A	0.552	0.601	0.875	0.660	0.552
	Encoder	0.657	0.683	0.882	0.712	0.657
	Pipe	0.608	0.659	0.884	0.720	0.608
	Pipe_A	0.556	0.602	0.874	0.656	0.556
P-glycoprotein substrate	Concat	0.851	0.850	0.849	0.850	0.851
	DNN	0.798	0.799	0.800	0.800	0.798
	DNN_A	0.804	0.804	0.805	0.805	0.804
	Encoder	0.866	0.867	0.868	0.868	0.866
	Pipe	0.840	0.840	0.841	0.841	0.840
	Pipe_A	0.824	0.824	0.824	0.824	0.824
Renal organic cation transporter	Concat	0.774	0.828	0.967	0.890	0.774
	DNN	0.649	0.710	0.949	0.784	0.649
	DNN_A	0.626	0.678	0.944	0.739	0.626
	Encoder	0.808	0.863	0.973	0.926	0.808
	Pipe	0.597	0.683	0.948	0.798	0.597
	Pipe_A	0.556	0.708	0.949	0.974	0.556
Total	Concat	0.749	0.786	0.911	0.824	0.757
	DNN	0.624	0.660	0.879	0.705	0.624
	DNN_A	0.636	0.670	0.882	0.717	0.636
	Encoder	0.760	0.788	0.915	0.821	0.760
	Pipe	0.682	0.719	0.903	0.767	0.682
	Pipe_A	0.612	0.637	0.880	0.672	0.612

**Table A2:** List of input properties in DrugBank dataset.

Input	Property Type
SMILES	String
Physiological charge	Int
Number of rings	Int
Rotatable bond count	Int
H bond acceptor count	Int
H bond donor count	Int
polarizability	Float
Molar Refractivity	Float
Monoisotopic weight	Float
Molecular weight	Float
Polar surface area	Float
LogP	Float
LogS	Float
Water Solubility	Float
Bioavailability	Boolean
Rule of five	Boolean
Veber’s rule	Boolean
MDDR-like rule	Boolean
Ghose filter	Boolean

**Table A3:** List of predicted properties in DrugBank dataset.

ADMET Property	TRUE	FALSE
Human intestinal absorption	TRUE	FALSE
Blood–Brain Barrier	TRUE	FALSE
Caco-2 permeable	TRUE	FALSE
P-glycoprotein substrate	Substrate	Non-substrate
P-glycoprotein inhibitor I	Inhibitor	Non-inhibitor
P-glycoprotein inhibitor II	Inhibitor	Non-inhibitor
Renal organic cation transporter	Inhibitor	Non-inhibitor
CYP450 2C9 substrate	Substrate	Non-substrate
CYP450 2D6 substrate	Substrate	Non-substrate
CYP450 3A4 substrate	Substrate	Non-substrate
CYP450 1A2 inhibitor	Inhibitor	Non-inhibitor
CYP450 2C9 inhibitor	Inhibitor	Non-inhibitor
CYP450 2D6 inhibitor	Inhibitor	Non-inhibitor
CYP450 2C19 inhibitor	Inhibitor	Non-inhibitor
CYP450 3A4 inhibitor	Inhibitor	Non-inhibitor
CYP450 inhibitory promiscuity	High CYP Inhibitory Promiscuity	Low CYP Inhibitory Promiscuity
Ames test	AMES toxic	Non-AMES toxic
Carcinogenicity	Carcinogens	Non-carcinogens
Biodegradation	Readily biodegradable	Not readily biodegradable
hERG inhibition (predictor I)	Week inhibitor	Strong inhibitor
hERG inhibition (predictor II)	Inhibitor	Non-inhibitor
